# Application of the Weighted-Incidence Syndromic Combination Antibiogram (WISCA) to guide the empiric antibiotic treatment of febrile neutropenia in oncological paediatric patients: experience from two paediatric hospitals in Northern Italy

**DOI:** 10.1186/s12941-024-00673-8

**Published:** 2024-02-15

**Authors:** Cecilia Liberati, Daniele Donà, Linda Maestri, Maria Grazia Petris, Elisa Barbieri, Elisa Gallo, Jacopo Gallocchio, Marta Pierobon, Elisabetta Calore, Annachiara Zin, Giulia Brigadoi, Marcello Mariani, Alessio Mesini, Carolina Saffioti, Elisabetta Ugolotti, Dario Gregori, Carlo Giaquinto, Elio Castagnola, Alessandra Biffi

**Affiliations:** 1 Division of Paediatric Infectious Diseases, Department for Women’s and Children’s Health, University of Padua, Padua, Italy; 2 Division of Paediatric Haematology, Oncology and Stem Cell Transplant, Department for Women’s and Children’s Health, University of Padua, Padua, Italy; 3 Unit of Biostatistics, Epidemiology and Public Health, Department of Cardiac, Thoracic, Vascular Sciences and Public Health, University of Padua, Padua, Italy; 4 Infectious Diseases Unit, Department of Pediatrics, IRCCS Istituto Giannina Gaslini, 16147 Genoa, Italy

**Keywords:** Febrile neutropenia, Empiric antibiotic treatment, Weighted-Incidence Syndromic Combination Antibiogram

## Abstract

**Background:**

Guidelines about febrile neutropenia in paediatric patients are not homogeneous; the best empiric treatment of this condition should be driven by local epidemiology. The Weighted-Incidence Syndromic Combination Antibiogram (WISCA) addresses the need for disease-specific local susceptibility evidence that could guide empiric antibiotic prescriptions based on outcome estimates of treatment regimens obtained as a weighted average of pathogen susceptibilities. This study developed a WISCA model to inform empirical antibiotic regimen selection for febrile neutropenia (FN) episodes in onco-haematological paediatric patients treated at two Italian paediatric tertiary centres.

**Methods:**

We included blood cultures from patients with a bloodstream infection and neutropenia admitted to the Paediatric Haematology-Oncology wards in Padua and Genoa Hospitals from 2016 to 2021. WISCAs were developed by estimating the coverage of 20 antibiotics as monotherapy and of 21 combined regimens with a Bayesian probability distribution.

**Results:**

We collected 350 blood cultures, including 196 g-negative and 154 g-positive bacteria. Considering the most used antibiotic combinations, such as piperacillin–tazobactam plus amikacin, the median coverage for the pool of bacteria collected in the study was 78%. When adding a glycopeptide, the median coverage increased to 89%, while the replacement of piperacillin–tazobactam with meropenem did not provide benefits. The developed WISCAs showed that no monotherapy offered an adequate coverage rate for the identified pathogens.

**Conclusions:**

The application of WISCA offers the possibility of maximizing the clinical utility of microbiological surveillance data derived from large hospitals to inform the choice of the best empiric treatment while contributing to spare broad-spectrum antibiotics.

**Supplementary Information:**

The online version contains supplementary material available at 10.1186/s12941-024-00673-8.

## Background

Febrile neutropenia (FN) is the most common complication of cancer chemotherapy in paediatric patients; a risk-stratification strategy is recommended to early detect those patients at greater risk of high morbidity and mortality [1, 2].

Bacteria are the most common causative pathogens, with multidrug-resistant organisms (MDROs) emerging as a significant threat in the management of neutropenic paediatric patients worldwide [3].

The strategy of administering broad-spectrum antibiotic therapy for managing FN has been introduced in the 60th of the last century and has reduced infection-related mortality, especially by Gram-negatives rods [4]. However, current recommendations on the drug regimens to be employed for treating FN are not straightforward due to the considerable heterogeneity in local epidemiology and resistance patterns. In clinically stable patients at low risk of resistant infections, monotherapy with an antipseudomonal non-carbapenem β-lactam plus β-lactamase inhibitor combination (e.g., piperacillin–tazobactam) or fourth-generation cephalosporin is recommended. In clinically unstable patients, recommendations suggest a first-line carbapenem, with or without a second anti-gram-negative agent, with or without a glycopeptide [1, 2].

Antimicrobial prescribing habits at different clinical centres may vary according to local practices and the prevalence of MDROs. The local pathogen prevalence is usually described by cumulative hospital antibiograms, which provide general information on the sensitivity of individual bacterial species or genera to certain antibiotics, with no further stratifications [5–7].

In response to these limitations, Hebert et al. developed Weighted-Incidence Syndromic Combination Antibiogram (WISCA) [8], a tool that addresses the need for disease-specific local susceptibility evidence that could guide empiric antibiotic prescriptions based on outcomes estimates of treatment regimens obtained as a weighted average of pathogen susceptibilities. In this way, WISCA guarantees the possibility of analysing different clinical and epidemiological aspects and could contribute to reducing antibiotic resistance.

In a previous study, we successfully applied a WISCA Bayesian model to guide empiric treatment in paediatric patients with urinary tract infections [9]. This study aims to assess the ability of WISCA to support and guide the selection of empiric antibiotic regimens for FN treatment in onco-haematological paediatric patients by comparing WISCAs developed using data from two northern Italy onco-haematological paediatric referral centres, and by comparing WISCA results with current recommendations.

## Materials and methods

### Study design and population

This is a multicentric, observational, retrospective study conducted at two Italian centers.

The onco-haematological paediatric ward of the Department for Women’s and Children’s Health at the University of Padua is a complex operative unit of the Paediatric Hospital of Padua, with a 19 + 1 beds ordinary ward, six beds in the stem cell transplant unit and ten beds in the outpatient facility. It accounts for about 750 admissions per year on average.

The onco-haematological paediatric ward of the IRCCS (*Istituto di Ricovero e Cura a Carattere Scientifico*) Giannina Gaslini children’s hospital in Genoa has 18 beds in ordinary ward, five beds in the bone marrow transplant unit and ten beds in the Day Hospital unit. It accounts for about an average of 640 admissions per year.

The study cohort included patients admitted with a microbiological diagnosis of bloodstream infection (BSI) and neutropenia. The study period ranged from January 1st, 2016, to December 31st, 2021.

### Inclusion criteria

A BSI episode was defined by the isolation of a pathogen in blood cultures, in children presenting with FN.

Criteria for blood cultures to be included are reported in the Additional file 1: S1.

Neutropenia was considered an absolute neutrophil count < 500/µL or < 1000/µL and rapidly declining [3, 10]. However, patients presenting with an uncontrolled or relapsed disease with blood prevalence of blasts were considered functionally neutropenic and then included.

### Data collection

We retrospectively reviewed the clinical documentation of patients identified by the positive blood cultures provided by the microbiology laboratory or captured by the hospital's electronic medical records. The following data were obtained from the identified BSI episodes: date of birth, sex, age at the time of the episode, date of positive blood culture, admitting hospital, haematological and/or oncological diagnosis, previous haematopoietic stem cell transplantation (HSCT), previous graft versus host disease (GvHD). The type of isolated pathogen (Gram stain and pathogen species) and antibiotic susceptibility test results were collected for positive blood cultures.

For both centres, bacteria isolates were identified by standard criteria, and susceptibility testing categories were classified according to the 2019 definitions by the European Committee on antimicrobial susceptibility testing (EUCAST) [11].

Tested antimicrobials were different between the years and between the two centres. When, in an antibiogram, a specific antibiotic was not tested, it was classified as not available and consequently did not weigh on the analysis. Untested antibiotics were however classified whenever a category was judged imputable (for example, all *Pseudomonas aeruginosa* were classified as resistant to ceftriaxone, and all methicillin-susceptible *Staphylococcus aureus* were considered susceptible to meropenem).

### WISCA model

WISCA model was developed as a decision tree (adapted from Bielicki et al. Additional file 1: S2) [12].

The WISCA probability of expected coverage for each antibiotic regimen was calculated considering the weighted probability of etiological pathogens and the probability of each pathogen being susceptible to the studied antibiotic or antibiotics.

WISCA, then, reflects the probability for an antibiotic to be appropriate for a specific infectious disease (FN in this case), lying between 0 (impossible for the antibiotic to be appropriate) to 1 (certainly appropriate antibiotic) [13].

Our study developed WISCA models (WISCAs) by estimating the coverage of 20 antibiotics as monotherapy and 21 combined antibiotics regimens based on the centre/international guidelines. Moreover, a second model considering only Gram-negative bacteria was created.

### Statistical analysis

To determine the odds of coverage by antibiotic treatment, we used a Bayesian logistic regression. In this context, pathogens and antibiotics were included in the model as random effects and age, sex, underlying pathology and HSCT occurrence were included in the model as fixed effects.

We tested the different coverage between centres with the Bayesian Leave-One-Out cross-validation and the evaluation of the differences between Expected Log-Predictive Densities (ELDPs) of the models with and without the centre effect. Posterior distributions were summarized using the median and the 95% *Bayesian Uncertainty Intervals* (BUIs*)* and the probability that the estimated coverage was at least 85%.

Categorical variables have been described as frequencies and percentages, while continuous variables have been expressed as median and interquartile range (IQR).

The incidence of each pathogen and its sensitivity to a given antibiotic treatment have been evaluated with an approach based on the WISCA tool.

To test whether there was a difference between the populations of the two centres, we applied a χ2 test or a Fisher exact test depending on the frequencies of the values.

Resulting WISCA values were expressed as median coverage provided, with associated upper and lower 95% BUIs and the probability that estimated antibiotic coverage was at least 85% *(p_85).* An empiric therapy was a priori considered acceptable if the median coverage was at least 75% of isolated pathogens. The probability that the coverage was at least 85% (p_85) was then calculated, considering BUIs.

Statistical analyses were conducted using R statistical software [14].

### Outcome

The primary outcome was the definition of the most appropriate antibiotic or combination of antibiotics to empirically treat neutropenic patients presenting with fever, applying a stratified WISCA model.

## Results

### Population characteristics

In the study period, 350 blood cultures were included.

Demographic and baseline features of the patients who tested positive for included blood cultures are shown in the Additional file 1: S3. The two cohorts of Padua and Genoa were homogeneous in terms of age groups, transplant status and GvHD distribution; differences were reported in sex (males were more frequent in Genoa) and underlying pathology (leukaemia was more frequent in Padua, solid tumours in Genoa, S3).

The difference in the model’s predictive performances with and without centre effect were non-significant, thus allowing us to pool the data of the two centres.

**Fig. 1:**
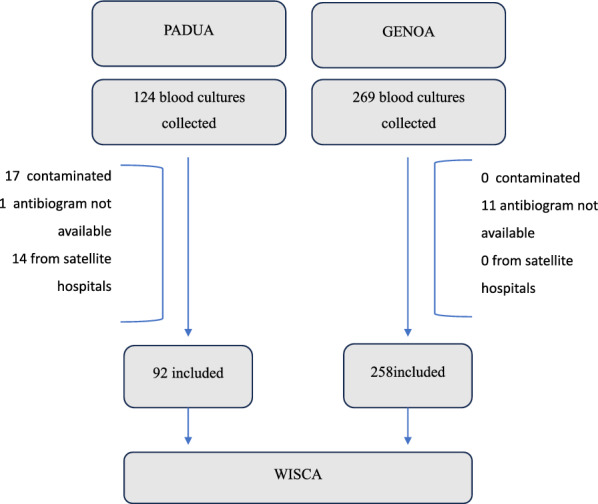
Flowchart of included blood cultures for the WISCA model

Included episodes are summarized in Fig. 1.

### Pathogen distribution

**Fig. 2:**
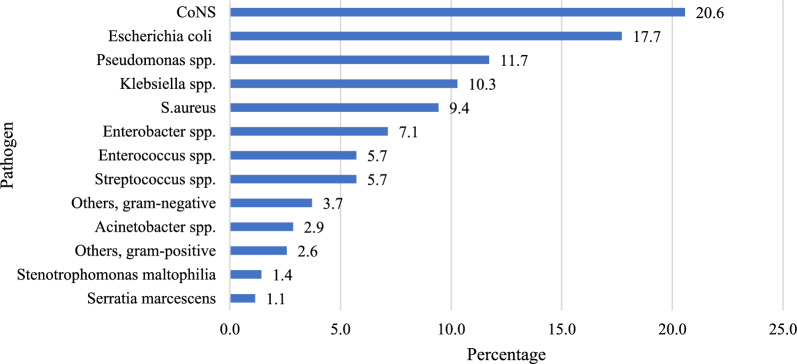
Pathogens causing BSI in the overall population

Data regarding the pathogen distribution and antimicrobial susceptibilities are summarized in Fig. 2 and Table 1.

**Table 1:** Incidence of resistances in the blood culture pool

Pathogen (N)	Resistance of concern	N (%)
Enterococcus spp. (20)	Ampicillin	10 (50)
	Vancomycin	1 (5)
*Staphylococcus aureus* (33)	Methicillin	1 (3)
*Coagulase-negative staphylococci* (72)	Methicillin	60 (83)
Gram negative bacteria (196)	Third-generation cephalosporins	59 (30)
	Carbapenems	9 (4.6)
	Piperacillin–tazobactam	30 (15.3)

### WISCA results

**Fig. 3:**
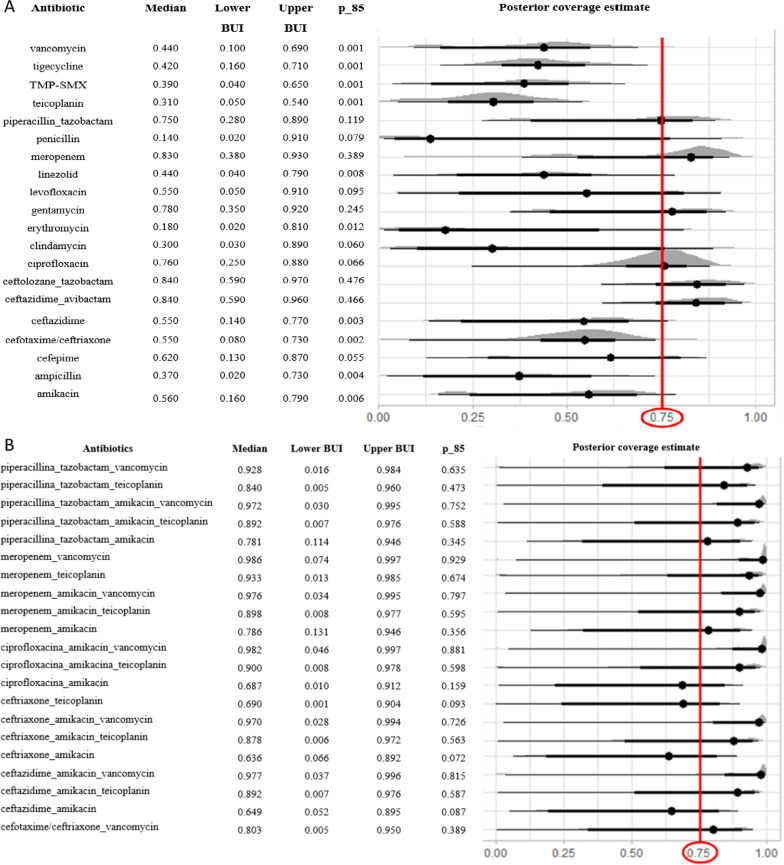
**A** Single antibiotics, **B** combined antibiotics: median coverage and the probability that the estimated coverage is at least 85% *(p_85).* Black Dots represent the median of the posterior distribution (first column) and the line of the associated 95% Bayesian Uncertainty Interval (BUI). The red line represents the threshold for the median coverage to be acceptable. TMP-SMX: trimethoprim–sulfamethoxazole

**Fig. 4:**
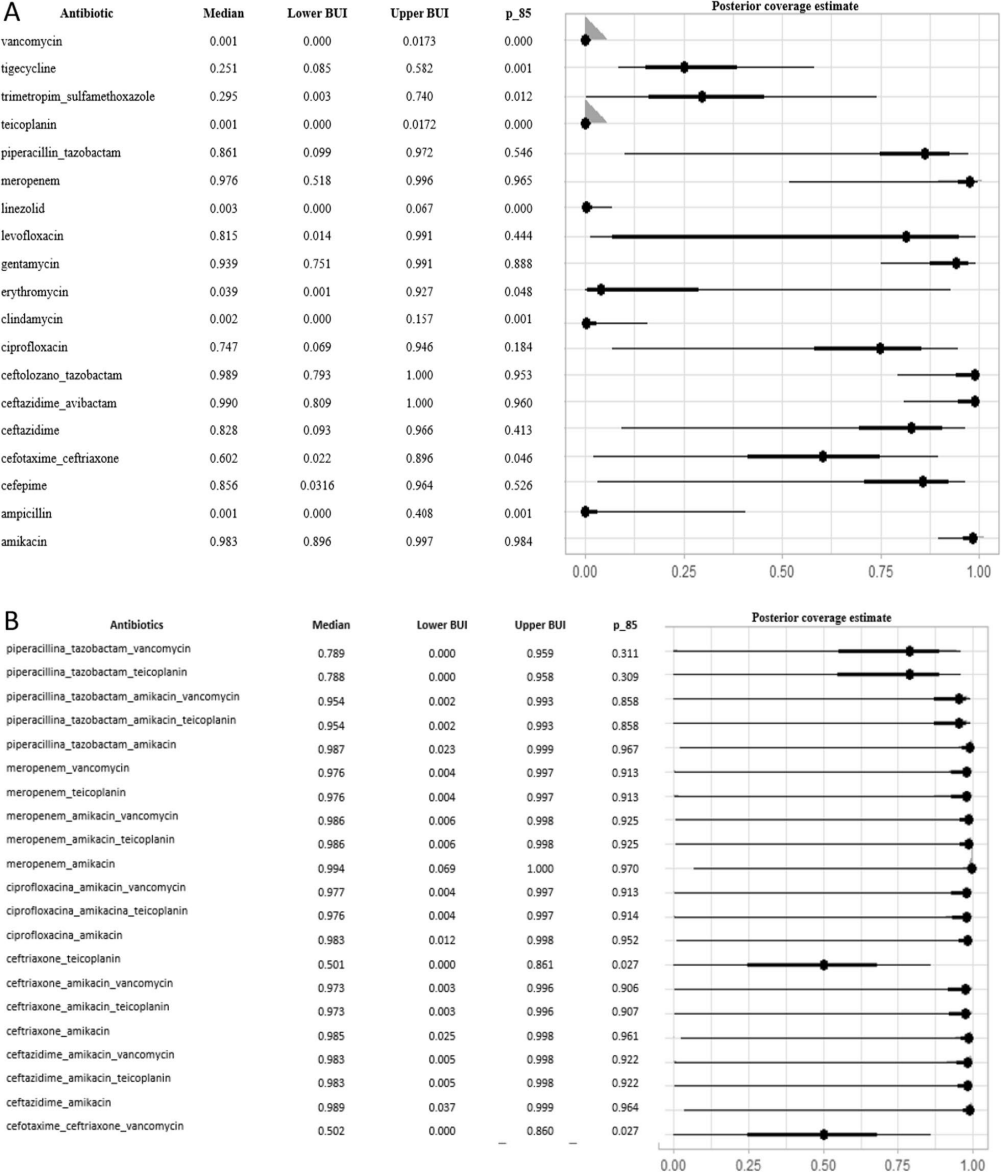
**A** Single antibiotics, **B** combined antibiotics, Gram-negative bacteria: Median coverage and the probability that the estimated coverage is at least 85% *(p_85).* Dots represent the median of the posterior distribution and line the associated 95% Bayesian Uncertainty Interval (BUI)

Twenty antibiotics were tested as monotherapies and 21 combined regimens were studied in the WISCA model for the overall pool of pathogens (Fig. 3) and for the only Gram-negative ones (Fig. 4).

### WISCA model with the overall population

For monotherapies, no antibiotic reached a posterior median coverage of 0.85, and only a few antibiotics (e.g. meropenem) included 0.85 in the confident interval (Fig. 3A). On the other hand, several combined regimens reached a good probability of providing at least 85% empirical antibiotic coverage (Fig. 3B).

The median coverage of piperacillin–tazobactam (PI-TZ) as monotherapy was 75%, while in the association PI-TZ-amikacin resulted to be 78%, and when adding the glycopeptide (vancomycin), it dramatically increased to 97%. Meropenem plus vancomycin reached a 98% median coverage, showing the unnecessity of a second gram-negative agent (i.e. amikacin) associated with a carbapenem.

Results are further reported in Table 2.

**Table 2:** Results from the WISCA model for all blood cultures included: percentage, BUI (Bayesian Uncertainty Intervals)

Guidelines for the management of FN	Estimated coverages of studied regimens from WISCA model
Use monotherapy with an antipseudomonal b-lactam, a fourth-generation cephalosporin or a carbapenem as empiric antibacterial therapy in pediatric high-risk FN (strong recommendation, high-quality evidence) Reserve addition of a second anti-Gram-negative agent or a glycopeptide for patients who are clinically unstable, when a resistant infection is suspected, or for centres with a high rate of resistant pathogens (strong recommendation, moderate-quality evidence)	Antipseudomonal b-lactam: 75% (0,28–0,89) Carbapenem: 83% (0,38–0,93) Anti-pseudomonal + second anti-gram negative: 78% (0,11–0,94) Carbapenem + second anti-gram negative: 78% (0,13–0,94) Anti-pseudomonal + second anti-gram negative + glycopeptide: 97% (0,03–0,99) Carbapenem + second anti-gram negative + glycopeptide: 97% (0,03–0,99)

On the left, recommendation for the management of febrile neutropenia, from Lehrnbecher et al., 2023 [1]

### WISCA model with gram-negative bacteria

In this second model, the analysis was performed considering only Gram-negative bacteria (196 blood cultures). Figure 4A describes the results for antibiotics in monotherapy, and Fig. 4B indicates the results of the 21 analysed combined antibiotics regimens. Monotherapy with PI-TZ showed a slightly inferior coverage (86%) compared with meropenem (98%); however, combining PI-TZ with amikacin, the coverage level increased up to 99%. There was no advantage in adding amikacin to meropenem.

## Discussion

WISCA has been previously used to study antibiotic coverages in different adult and paediatric infectious syndromes [12, 13, 15–17]. To our knowledge, this is the first study developing a WISCA model to guide the empirical choice of the most suitable antibiotic empiric therapy in onco-haematological paediatric patients. The secondary immunodeficiency and the high exposure of patients to previous antibiotic treatments pose a challenge for severe infections, possibly due to MDR organisms, making this population a unique epidemiological setting where empirical therapies need to be optimized [18, 19].

Genoa collected many more blood cultures compared to Padua, this is probably due to the different diagnostic strategies for children presenting with FN. In particular, Genoa provided more solid tumours in its population. As the epidemiology of tumours does not change in the same region, we believe this is the consequence of an increased diagnostic capacity, especially for catheter-related infections, caused by CoNS, which are the majority of episodes in solid tumours children in Genoa centre.

The primary objective of this study was to assess the ability of a Bayesian WISCA model to estimate antibiotic treatment appropriateness, which is not intended as 100% coverage, but as an acceptable compromise between pathogen coverage and responsible antimicrobial prescribing practice. Our study showed that, despite recommendations [1, 2], none of the monotherapies offered an adequate coverage rate for the identified pathogens; indeed, both centres are not currently using monotherapies to manage FN. However, combination therapies considerably increased the median coverage rates. According to the principle that led to the strategy of empirical therapy (early treatment of Gram-negative bacteraemia to reduce mortality [4]) the key to reaching the optimal coverage rate was the association of an anti-pseudomonas molecule with a second gram-negative agent, as amikacin. The association with a glycopeptide further increased the coverage rate. In our settings this may have a limited clinical relevance, but it is probably the result of the high incidence of methicillin-resistant CoNS (Table 1), while methicillin-resistant *S. aureus* was found only once. However, the glycopeptide-containing combination could be useful in patients colonized by methicillin-resistant *S. aureus* that present a significantly higher risk of *S. aureus* bacteraemia that would not be adequately treated by anti-Gram-negative antibiotics (such as PI-TZ) [3]. Teicoplanin (in all combinations) performed worse than vancomycin. This is due to the limited number of CoNS tested for teicoplanin, which is not routinary tested.

Our study confirms the reliability of the combination of PI–TZ–amikacin, resulting in a median coverage of 98% when focusing exclusively on gram-negative bacteria. Considering the entire pool of isolated bacteria, including both gram-positive and gram-negative pathogens, PI–TZ–amikacin provides a coverage of 78%, which increases to 97% after the addition of vancomycin.

Uncertainty intervals, overall, were quite large, due to the small sample size.

In certain situations, such as certain combinations (e.g., ciprofloxacin-amikacin), they appear to perform worse than monotherapy (ciprofloxacin alone). This results in a decrease in sample size when considering cultures with both drugs tested.

Paediatric BSI is uncommon in onco-haematological patients [20], and data are limited even in a six-year, bicentric study. The small-sample limitation is a known issue when applying WISCA: in a previous study, Bielicki et al. in 2016 used WISCA to evaluate five empirical antibiotic regimens for paediatric BSI, using pooled data from 19 hospitals to overcome sample-narrowness. Although statistical significance was achieved, the results could not be generalized as the epidemiology of BSI was not overlapping between centres [12]. This highlights the pathogen temporal and geographical variability as intrinsic characteristics of infectious syndrome epidemiology, limiting the utility of applying data gathered from heterogeneous cohorts, but confirms the recommendation of antibiotic choices based on local epidemiological data [1, 2].

Another way to reach significance when evaluating antibiotic coverage is to limit the analysis to a few regimens and a few, most prevalent, pathogen isolates. This approach has been successfully used to study coverage of third-generation cephalosporins and meropenem toward causative pathogens of paediatric BSI from 23 countries [17]; however, these results cannot be applied to a local level to drive empiric prescribing, with limited clinical usefulness. Indeed, for local adaptation, it would be necessary to identify that local pathogen epidemiology and susceptibility patterns are homogeneous to the pooled data from where WISCA was calculated.

The analysis in this study was not restricted, including even once-found bacteria (which will, however, “weight less”), as onco-haematological patients are a high-risk population, and empiric coverage must consider even uncommon aetiologies, as sepsis in neutropenic patients may be a life-threatening event. The same global approach has been used to study critical care infections in adults by Randhawa et al. [15]. However, in neutropenic patients, infections with Gram-negative bacteria have a poorer outcome and are associated with increased adverse events [21]. Thus, this study developed a second WISCA model including only Gram-negative bacteria, considering the possibility of targeting Gram-positive bacteria (as CoNS) in clinically stable patients when cultures turn back positive. This strategy is currently adopted in Genoa Hospital. The restriction of glycopeptide use in paediatric cancer patients with FN resulted safe in an observational study [22].

Further, we decided not to restrict the analysis to a few antimicrobial regimens, as drug prescribing in onco-haematological patients is often challenging due to possible organ dysfunction, allergies, drug-to-drug interactions, fluid overload, and venous catheter incompatibilities. We then decided to analyse all monotherapies and combinations potentially used, considering even those antibiotics that are not considered first-line molecules in children, such as ciprofloxacin.

Another limitation of the WISCA application in our study is the lack of correlation between infective pathogens and infection outcomes (e.g., mortality, PICU admission). Those data would allow a WISCA model to estimate regimens with expected maximum clinical concordance and, therefore, the most significant potential impact. This strategy has not yet been adopted in the reported WISCA study.

Lastly, when applying empiric antibiotic therapy, an “acceptable” cut-off of coverage is usually self-estimated according to clinicians’ and microbiologists’ experience. We used an 85% estimated median coverage rate to define a regimen “appropriate”, which overlaps with the study by Randhawa et al. on critical care infections. However, many clinicians could have a preference for antibiotic regimens perceived to have a coverage of 90% or more [23].

## Conclusions

The Bayesian WISCA provides an innovative approach to pool information from different sources about a specific infectious syndrome compared to standard hospital antibiograms. WISCA gave tailored information about the empiric antimicrobial therapies for paediatric patients with fever and neutropenia. Efforts to include more significant numbers of cultures and clinical outcomes may overcome the statistical limitations of this approach.

The application of WISCA in a multicentre study offers the possibility of maximising the clinical utility of microbiological surveillance data derived from larger hospitals to inform the selection of the most appropriate empirical antibiotic therapy also for other minor hospital settings in the same area while contributing to spare broad-spectrum antibiotics and increasing confidence in the selection of narrow-spectrum regimens.

### Supplementary Information

**Additional file 1.** Supplementary methods, results.

## Data Availability

The datasets used and/or analysed during the current study are available from the corresponding author on reasonable request.

## Abbreviations

BSI: Bloodstream infection
BUI: Bayesian Uncertainty Interval
CoNS: Coagulase Negative Staphylococci
EUCAST: European Committee on antimicrobial susceptibility testing
FN: Febrile neutropenia
GvHD: Graft versus host disease
HSCT: Haematopoietic stem cell transplantation
IQR: Interquartile ranges
MDROs: Multidrug resistance Organisms
WISCA: Weighted-Incidence Syndromic Combination Antibiogram
PI-TZ: Piperacillin–tazobactam
TMP-SMX: Trimethoprim–sulfamethoxazole
