# Supplementary material for: Application of the Weighted-Incidence Syndromic Combination Antibiogram (WISCA) to guide the empiric antibiotic treatment of febrile neutropenia in oncological paediatric patients: experience from two paediatric hospitals in Northern Italy
Source: Ann Clin Microbiol Antimicrob. 2024 Feb 15;23:16. doi: 10.1186/s12941-024-00673-8 (PMC10870518; doi:10.1186/s12941-024-00673-8)
Supplement: Supplementary file 1 — Additional file 1. Supplementary methods, results. [file 12941_2024_673_MOESM1_ESM.docx]

Additional file

**S1 – Criteria of inclusion of bloodstream infection (BSI) in patients presenting with fever and neutropenia:**

- A BSI episode was defined by the microbiological isolation of a pathogen in blood cultures.
- Only the first culture was considered if a pathogen was isolated in repeated cultures within the same infectious episode.
- In the case of isolating coagulase-negative staphylococci (CoNS) or other pathogens considered possible contaminants (micrococcus spp, streptococci viridans, bacillus spp, proteus, corynebacterium), the episode was included only if at least two separated blood cultures resulted positive for the same microorganism. If these organisms grew together with other bacteria considered pathogens in blood culture, it was considered a poly-microbial infection only if the suspected contaminant was isolated more than once.
- If the same patient presented with two or more different episodes of BSI, it was taken into account more than once.
- The isolation of the same pathogen from blood cultures for a patient was considered within the same episode if there were less than 20 days between cultures. If cultures remained negative for more than 20 days and the same pathogen was isolated, it was considered a second episode.
- Blood cultures with identified pathogens for which an antibiogram was not available were excluded

We did not have information about the possibility of secondary bacteremia. While

it is true that the site of infection is crucial in choosing appropriate

therapy, the occurrence of localized infections is relatively minor,

compared to primary bloodstream infections (excluding central line-associated BSI). In the context of empiric treatments, the antibiogram is not influenced by the source of infections, and therefore, it should not impact the

analysis.

**S2** – Decisional tree of WISCA model (adapted from Bielicki et al.)^12^

**
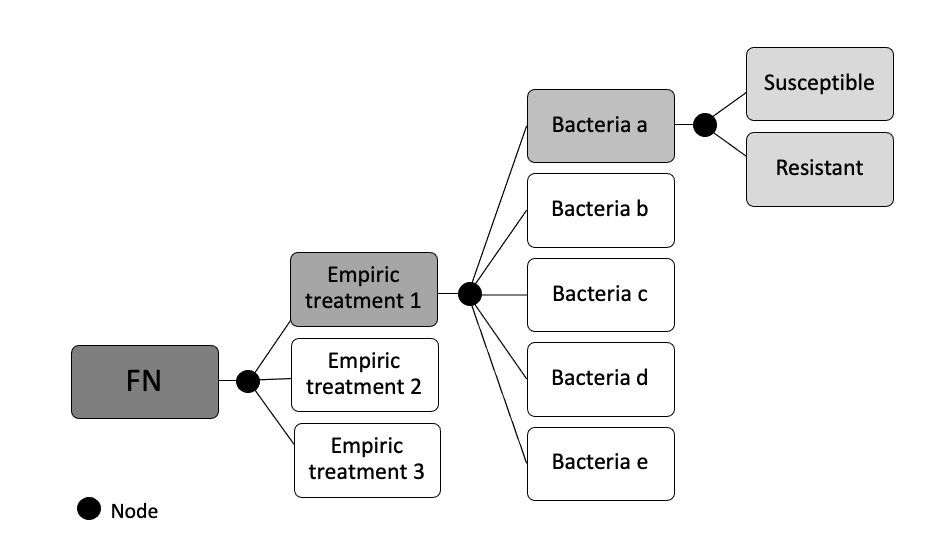
**

The first node (circle) represents the clinical decision to initiate empiric treatment in the case of FN, followed by regimens of interest of the study. The second node represents the proportion of episodes accounted for by each pathogen, and the third node represents the proportion of episodes susceptible to that regimen.

**S3** – Demographic characteristics of included patients with p-values referred to the overall cohort stratified by centers (Genoa and Padua).

| **Charateristics** | **N = 350** | **GENOA**  **N = 258 (74%)** | **PADUA**  **N = 92 (26%)** | **p-value** |
| --- | --- | --- | --- | --- |
| **Age (median (IQr))** | 8.6 (3.3 – 14) | 7.9 (2.7 – 13.1) | 10.3 (5.9 – 17.7) |  |
| **Age group** |  |  |  | 0.140 |
| < 3 years | 75 (21%) | 63 (24%) | 12 (13%) |  |
| 3 - 5 years | 53 (15%) | 42 (16%) | 11 (12%) |  |
| 6 - 8 years | 53 (15%) | 38 (15%) | 15 (16%) |  |
| 9 - 14 years | 99 (28%) | 68 (26%) | 31 (34%) |  |
| 15 - 19 years | 58 (17%) | 39 (15%) | 19 (21%) |  |
| ≥ 20 years | 12 (4%) | 8 (4%) | 4 (4%) |  |
| **Sex** |  |  |  | 0.003 |
| Female | 130 (37%) | 84 (33%) | 46 (50%) |  |
| Male | 220 (63%) | 174 (67%) | 46 (50%) |  |
| **Gram** |  |  |  | 0.067 |
| Gram - | 196 (56%) | 137 (53%) | 59 (64%) |  |
| Gram + | 154 (44%) | 121 (47%) | 33 (36%) |  |
| **HSCT** |  |  |  | 0.100 |
| No | 226 (65%) | 173 (67%) | 53 (58%) |  |
| Yes | 124 (35%) | 85 (33%) | 39 (42%) |  |
| **GvHD** |  |  |  | >0.900 |
| No | 79 (64%) | 54 (64%) | 25 (64%) |  |
| Yes | 45 (36%) | 31 (36%) | 14 (36%) |  |
| **Underlying pathology** |  |  |  | <0.001 |
| Other | 26 (8%) | 21 (8%) | 5 (5%) |  |
| Aplastic anaemia | 43 (12%) | 32 (12%) | 11 (12%) |  |
| Leukaemia | 193 (55%) | 126 (49%) | 67 (73%) |  |
| Lymphoma | 17 (5%) | 14 (6%) | 3 (3%) |  |
| Solid tumor | 71 (20%) | 65 (25%) | 6 (7%) |  |

GvHD: Graft versus host disease; HSCT: haematopoietic stem cell transplantation; IQr: interquartile range.

**S4** - Frequency of pathogenic species stratified by centres.

| **Total**  **N = 350** | **Pathogen** | **GENOA**  **N = 258 (74%)** | **PADUA**  **N = 92 (26%)** |
| --- | --- | --- | --- |
| **Gram –**  **N = 196 (56%)*** |  | N= 137 | N = 59 |
| 62 (17.7%)^§^ | Escherichia coli | 40 (15.5%) | 22 (23.9%) |
| 33 (9.4%)^§^ | Pseudomonas aeruginosa | 23 (8.9%) | 10 (10.9%) |
| 30 (8.6%)^§^ | Klebsiella pneumoniae | 19 (7.4%) | 11 (12%) |
| 23 (6.6%) | Enterobacter cloacae | 13 (5%) | 10 (10.9%) |
| 10 (2.9%) | Acinetobacter spp. | 9 (3.5%) | 1 (1.1%) |
| 8 (2.3%) | Pseudomonas spp. | 8 (3%) | 0 (0%) |
| 6 (1.7%) | Klebsiella oxytoca | 6 (2.3%) | 0 (0%) |
| 5 (1.4%) | Stenotrophomonas maltophilia | 5 (1.9%) | 0 (0%) |
| 4 (1.1%) | Serratia marcescens | 1 (0.4%) | 3 (3.3%) |
| 2 (0.6%) | Campylobacter jejuni/coli | 1 (0.4%) | 1 (1.1%) |
| 2 (0.6%) | Citrobacter koseri | 2 (0.8%) | 0 (0%) |
| 2 (0.6%) | Enterobacter hormaechei | 2 (0.8%) | 0 (0%) |
| 2 (0.6%) | Moraxella spp. | 2 (0.8%) | 0 (0%) |
| 1 (0.3%) | Aeromonas sobria | 0 (0%) | 1 (1.1%) |
| 1 (0.3%) | Capnocytophaga sputigena | 1 (0.4%) | 0 (0%) |
| 1 (0.3%) | Haemophilus influenzae | 1 (0.4%) | 0 (0%) |
| 1 (0.3%) | Neisseria mucosa | 1 (0.4%) | 0 (0%) |
| 1 (0.3%) | Ochrobactrum anthropi | 1 (0.4%) | 0 (0%) |
| 1 (0.3%) | Proteus spp. | 1 (0.4%) | 0 (0%) |
| 1 (0.3%) | Salmonella spp. | 1 (0.4%) | 0 (0%) |
| **Gram +**  **N = 154 (44%)*** |  | N = 121 | N=33 |
| 72 (20.6%)^ | Coagulase-negative Staphylococcus (CoNS) | 58 (22.5%) | 14 (15.2%) |
| *60* | *Methicillin-Resistant* |  |  |
| *12* | *Methicillin-Sensible* |  |  |
| 33 (9.4%) | Staphylococcus aureus | 28 (10.9%) | 5 (5.4%) |
| *1* | *Methicillin-Resistant* |  |  |
| *32* | *Methicillin-Sensible* |  |  |
| 20 (6%) | Streptococcus spp. | 16 (6.2%) | 4 (4.3%) |
| 14 (4%) | Enterococcus faecium | 8 (3%) | 6 (6.5%) |
| 6 (1.7%) | Enterococcus faecalis | 5 (1.9%) | 1 (1.1%) |
| 2 (0.6%) | Bacillus cereus | 0 (0%) | 2 (2.2%) |
| 2 (0.6%) | Rothia mucilaginosa | 2 (0.8%) | 0 (0%) |
| 1 (0.3%) | Brevibacterium casei | 1 (0.4%) | 0 (0%) |
| 1 (0.3%) | Clostridium tertium | 1 (0.4%) | 0 (0%) |
| 1 (0.3%) | Corynebacterium aurimucosum | 1 (0.4%) | 0 (0%) |
| 1 (0.3%) | Micrococcus luteus | 1 (0.4%) | 0 (0%) |
| 1 (0.3%) | Paenibacillus | 1 (0.4%) | 0 (0%) |

* the difference in gram-negative and gram-positive pathogens distribution was not significant between the two cohorts, p value 0.0673.

^ the difference in CoNS distribution was not significant between the two cohorts, p value 0.1389.

^§^ the difference in E.coli, P.aeruginosa, K.pneumoniae incidence in the two cohorts was not significant (p values, respectively, 0.0697, 0.5817, 0.1767).
